# Safety Considerations for Humans and Other Vertebrates Regarding Agricultural Uses of Externally Applied RNA Molecules

**DOI:** 10.3389/fpls.2020.00407

**Published:** 2020-04-23

**Authors:** Thais B. Rodrigues, Jay S. Petrick

**Affiliations:** ^1^GreenLight Biosciences, Inc., Medford, NC, United States; ^2^Bayer Crop Science, Chesterfield, MO, United States

**Keywords:** double-stranded RNA, biopesticide, human safety, RNA interference, sprayable dsRNA

## Abstract

The potential of double-stranded RNAs (dsRNAs) for use as topical biopesticides in agriculture was recently discussed during an OECD (Organisation for Economic Co-operation and Development) Conference on RNA interference (RNAi)-based pesticides. Several topics were presented and these covered different aspects of RNAi technology, its application, and its potential effects on target and non-target organisms (including both mammals and non-mammals). This review presents information relating to RNAi mechanisms in vertebrates, the history of safe RNA consumption, the biological barriers that contribute to the safety of its consumption, and effects related to humans and other vertebrates as discussed during the conference. We also review literature related to vertebrates exposed to RNA molecules and further consider human health safety assessments of RNAi-based biopesticides. This includes possible routes of exposure other than the ingestion of potential residual material in food and water (such as dermal and inhalation exposures during application in the field), the implications of different types of formulations and RNA structures, and the possibility of non-specific effects such as the activation of the innate immune system or saturation of the RNAi machinery.

## Introduction and Purpose of This Review Publication

The OECD (Organisation for Economic Co-operation and Development) Conference on RNA interference (RNAi)-based pesticides provided an overview of the current state of the art related to externally applied double-stranded RNA (dsRNA)-based products, also called exogenously or topically applied dsRNA. The purpose of the meeting was to facilitate exchanges between policymakers, academia, and industry on the implications of these products in health, the environment, and regulation, and to solicit inputs and recommendations based on these discussions^1^. The Conference was divided into three sessions. The first session provided a summary of the state-of-the-art of this technology: molecular mechanism and relevant RNAi pathways, current understanding of RNAi in different organisms, specificity level and its potential impact on non-target species, as well as the challenges associated with achieving RNAi efficacy in insects. The second session dealt with factors related to variation in insect responsiveness to environmental dsRNA, the environmental dissipation of dsRNA molecules in soil, water and plants, and the aspects of RNA-seq (RNA-sequencing) to validate RNAi data. It also presented an overview of available literature on the possible effects of exogenous dsRNA in humans and other vertebrates and addressed the regulatory experience with dsRNA applications in human therapeutics.

The third and final session summarized regulatory and risk assessment experiences whereby presenters identified “problem formulation” as a regulatory pathway similar to that for other biologically based active ingredients, explored better decision making ideas, and considered a case-by-case approach to assess ecological risks. It also reviewed the use of formulations to overcome hurdles for controlling insects that are recalcitrant to dsRNA and the experience of experts on the regulation of RNAi-based genetically engineered (GE) crops. To date, there is no precedent of externally applied RNAi-based products approved by regulatory agencies, even though the technology has already been developed and approved for genetically engineered (GE) crops. Although there are substantial differences between RNAi-based GE crops and biopesticides that impact the risk assessment of both products differently (such as dsRNA exposure duration, route, and dose), the experience of experts on regulation of RNAi-based GE crops provides helpful information on the safety of this new technology, guides interpretations of new studies, and supports regulatory requirements.

This report focuses on the third OECD meeting session that presented the possible effects of RNAi-based biopesticides in humans and other vertebrates. To introduce RNAi technology and its mode of action, the RNAi mechanism and main gene silencing pathways are described. RNAi technology comes as an alternative crop protection solution that enables a more specific, safer, and environmentally friendly tool for agricultural production (Bachman et al., 2013, 2016; Dubelman et al., 2014; Petrick et al., 2015; Parker et al., 2019). Even though RNAi technology has been studied for more than a decade and has been developed for use in GE crops (Baum et al., 2007; Bolognesi et al., 2012) and biopesticide products (Joga et al., 2016; McLoughlin et al., 2018), most of the available data concerning mammalian risk assessment comes from learnings within therapeutic research and studies developed to support the safety of GE crops. Because of its relevance to the topic and the ability to translate the data to RNAi-based biopesticides, this review presents and discusses key RNA studies in the GE crop literature and the history of safe consumption of dsRNA by humans and animals as well as describes the biological barriers responsible for the lack of response to exogenous RNA in these organisms. Additional considerations for human health specifically important for RNAi-based products were also discussed during the OECD Conference and are further discussed herein. The session included the implications of different types of formulations or forms of RNA structures, considered the other routes of exposure of a sprayable product during the product application (dermal and inhalation) and discussed the possibility of non-specific effects such as the activation of the innate immune system or RNAi machinery saturation.

## Overview of RNAi Mechanisms in Vertebrates

RNAi is a post-transcriptional process ubiquitous in eukaryotes that results in degradation or translational suppression of specific messenger RNA (mRNA) molecules, leading to a reduction in protein production. Suppression of the gene product occurs inside the cells and is initiated from either exogenous dsRNA or RNA molecules originating internally from the nucleus (reviewed by Carthew and Sontheimer, 2009). Herein, we discuss two key RNAi pathways that can be leveraged for gene expression regulation in an agricultural setting, small interfering RNA (siRNA) and microRNA (miRNA).

The siRNAs are short duplex sequences of ∼21–23 nucleotides (nt) (Elbashir et al., 2001) derived from the cleavage of both endogenous and exogenous dsRNAs. The dsRNA is specifically processed in the cellular cytoplasm by Dicer and Dicer-like proteins, which are members of the RNase III family of nucleases (Macrae et al., 2006). The duplex siRNAs are unwound by a helicase (yet to be identified) separating both sense and antisense strands (reviewed by Bartel, 2004). The siRNA strand with the less stable base pair at its 5′ end in the duplex, called the antisense strand, is loaded into a multiprotein complex called RISC (RNAi-Induced Silencing Complex) and the sense strand is degraded (reviewed by Winter et al., 2009). The antisense strand acts as a guide that recognizes the target mRNA by complementary base-pairing and Argonaute, a component of RISC, degrades the mRNA, leading to gene product suppression (Martinez et al., 2002; Khvorova et al., 2003; Winter et al., 2009).

The miRNAs are processed from their long dsRNA precursors into short ssRNA molecules that feed into an analogous pathway to that described above for siRNAs, leading to gene product suppression of complementary mRNA targets. This can occur through both translational suppression (Olsen and Ambros, 1999; Hutvágner and Zamore, 2002; Georgantas et al., 2007) and mRNA cleavage (Mansfield et al., 2004; Yekta et al., 2004). In animals, the miRNA precursors, termed primary miRNAs (pri-miRNAs) have a complex structure consisting of self-complementary sequences separated by a short non-complementary sequence. These molecules fold into intramolecular hairpins and often contain a small number of mismatched bases that create bubble-like structures (Cullen, 2004). The pri-miRNA is processed in the nucleus by Drosha, another enzyme member of the RNase III family, generating ∼65 nt stem-loop intermediate known as miRNA precursor (pre-miRNA) (Lee et al., 2003). The pre-miRNAs are transported to the cytoplasm through the nuclear export receptor Exportin-5 (Expo-5) and processed by Dicer, generating a ∼20 nt mature miRNA which is similar in both structure and function to the siRNA duplexes (Hutvágner and Zamore, 2002). Despite the similarity with siRNA duplexes, each miRNA hairpin precursor molecule produces a single miRNA duplex, whereas each long dsRNA produces multiple siRNAs. As with processed siRNAs, miRNAs are unwound by helicases, the sense strand degraded, and the antisense strand incorporated into RISC (Khvorova et al., 2003). The miRNA guides RISC to the mRNA target and the complex and its component Argonaute induces mRNA degradation or translational repression (Meister and Tuschl, 2004). Different than the siRNA mechanism where all bases generally contribute to its target specificity, complementarity between a miRNA and its target is usually partial, meaning it can regulate transcripts with limited complementarity to the antisense strand of the miRNA duplex (Lam et al., 2015). However, the high complementarity with a contiguous stretch of at least six nucleotides beginning at position two of the 5′ end of the miRNA, the seed region, has shown to be important for miRNA induced gene regulation (Jackson et al., 2006). Pairing exclusively with the seed region is not enough to induce the target mRNA cleavage but may result in translational pause (Mullany et al., 2016).

## History of Safe RNA Consumption

As presented above, RNAi is a highly conserved mechanism in eukaryotes for gene expression regulation. Small RNAs such as siRNAs and miRNAs are ubiquitous in commonly consumed plant and animal-derived foods. A number of small RNAs with perfect complementarity to human and animal genomes and transcriptomes have been identified in crops widely consumed globally, such as soybean, corn, and rice (Ivashuta et al., 2009). Corn specifically contains endogenous small RNAs that match approximately 450–2300 unique protein coding RNA transcripts in rat, mouse, and human (Petrick et al., 2016a). Fresh market fruits and vegetables also contain small RNAs with sequence complementarity to human genes. Most of these RNAs are likely derived from their genome, but a portion may also originate from plant viruses, which is an exogenous source (Frizzi et al., 2014). The established history of safe consumption of both exogenous and endogenous RNA molecules in food and feed that have 100% sequence complementarity to human and animal transcripts suggests that there is no negative biological effect of ingested RNAs, and supports safety of these molecules for use as agricultural active ingredients (Petrick et al., 2013; Frizzi et al., 2014).

In addition to the safe consumption of conventional crops, fruits, and vegetables, RNAi-mediated plant phenotypes have been found in many domesticated crops and have been used in approved biotech crop traits for more than two decades (reviewed by Petrick et al., 2013; Sherman et al., 2015). Some examples of RNAi-based traits include RNAi-mediated resistance to the ipomovirus CBSUV in cassava (Yadav et al., 2011), Innate^TM^ potatoes with reduced acrylamide production potential and blackspot bruise resistance (Simplot, 2014) and recently approved by US EPA, MON 87411, a corn plant expressing the DvSnf7 RNA PIP (plant-incorporated protectant), encoding a dsRNA that confers RNAi-mediated control of corn rootworms (Bachman et al., 2016). In support of the human and mammalian safety assessment of MON 87411, Petrick et al. (2016a, b) performed a 28-day repeat dose toxicity study in mice with DvSnf7 RNA and did not observe any effects on body weights, food consumption, clinical observations, clinical chemistry, hematology, gross pathology, or histopathology endpoints. They concluded there are no adverse health effects in mammals administered an insect active RNA molecule at doses millions to billions of times higher than anticipated human exposures (Petrick et al., 2016a, b).

## Biological Barriers

Vertebrates consume RNA molecules with every meal through foods of plant, animal, or fungal origin. This includes dsRNAs of various lengths that, based on sequence, would be capable of initiating the RNAi pathway if they were to reach a target cell. As presented previously, there are many such dietary dsRNAs that have sequence identity to genes in consuming vertebrates (Heisel et al., 2008; Ivashuta et al., 2009; Jensen et al., 2013; Frizzi et al., 2014; Petrick et al., 2016a) and without biological barriers protecting such organisms from these RNAs, every bite of every meal would present a potential source of regulation of protein production. Biological barriers faced by ingested RNAs (reviewed by O’Neill et al., 2011; Petrick et al., 2013) ensure homeostasis after RNA ingestion rather than a potential for these RNAs to impact gene expression in the consuming organism.

Following ingestion, food is chewed, and during this process, dietary RNA molecules are presented with nucleases in the saliva (Park et al., 2006) that begin to break down ingested RNA. As food passes the “oral phase” of digestion and is swallowed, dietary RNAs reach the stomach, which presents a digestive environment that results in extensive digestion of ingested nucleic acids (Petrick et al., 2013; Huang et al., 2018). This digestion occurs through a low pH environment in the stomach and leads to denaturation and depurination of nucleic acids followed by hydrolytic fragmentation (O’Neill et al., 2011). From the stomach, RNAs in partially digested food reach the small intestine, containing a digestive milieu that includes nucleases and degradative enzymes secreted from the pancreas that degrade nucleic acids, further digesting ingested RNA molecules into shorter nucleotides (O’Neill et al., 2011). From the small intestine, RNAs can transit through the gastrointestinal tract and be eliminated in the feces or in some cases smaller nucleic acids may be absorbed into the gastrointestinal epithelium and undergo further distribution throughout the body.

For an exogenous RNA to undergo absorption from the lumen of the intestinal tract, the RNA must cross a series of cellular membrane barriers. This includes the apical and distal membranes of the gastrointestinal (GI) epithelial cells that a given RNA must transverse and if such an RNA is to reach the bloodstream, both apical and distal membranes of the vascular endothelium. This must also occur for an RNA to cross into a distal tissue, e.g., the RNA would have to again leave the vasculature through the endothelium and cross the epithelium of another tissue to have the potential to regulate gene expression in that tissue. Each of these cellular membrane layers is a lipid bilayer that is highly impermeable to polar macromolecules such as RNA (Gilmore et al., 2004; Petrick et al., 2013). Any RNA reaching the interior of a cell must also escape endosomes, cellular compartments that sequester a majority of RNA molecules that enter a cell (e.g., 98–99%), posing a significant barrier to efficacy of RNA therapeutics (White, 2008; Gilleron et al., 2013). This would also present a significant barrier to any ingested RNAs that undergo cellular uptake. To further complicate the transit of dietary RNA through the systemic circulation, nucleases in the blood serve to degrade RNA molecules (Houck, 1958; Layzer et al., 2004; White, 2008; Christensen et al., 2013). In addition, RNA is cleared rapidly from the bloodstream via renal elimination (White, 2008; Molitoris et al., 2009; Thompson et al., 2012). These barriers are reviewed and pictorially represented in the peer reviewed scientific literature (Petrick et al., 2013). The collective series of barriers described above and summarized in Table 1 presents a formidable challenge to therapeutic developers, necessitating chemical stabilization of RNA therapeutics and their formulation in lipid and other delivery systems to enable escape from these barriers and to increase their resistance to degradation (O’Neill et al., 2011; Forbes and Peppas, 2012).

**TABLE 1:** List of significant biological barriers to ingested RNAs in humans.

**Biological Barriers**		
**Gastrointestinal**	**Systemic**	**Cellular**
Saliva	Vascular endothelium	Cellular and nuclear membrane barriers
Stomach acids/Digestive enzymes	Serum nucleases	Endosomes and lysosomes
Pancreatic nucleases	Renal filtration and elimination	Sufficient sequence identity
GI epithelium and tight junctions	Tissue epithelium	Gene target accessibility

The efficacy of these barriers can be observed in therapeutic studies in which injected RNA drugs that are unformulated are both readily degraded and rapidly excreted (Tillman et al., 2008; White, 2008; Molitoris et al., 2009; Thompson et al., 2012; Christensen et al., 2013). Observations of limited RNA uptake due to biological barriers have been made with ingested/orally administered RNA molecules in both dietary and therapeutic settings (Tillman et al., 2008; Dickinson et al., 2013; Snow et al., 2013; Huang et al., 2018), a subject that will be discussed in greater detail within this manuscript.

## Published Mammalian Studies

The above discussion on biological barriers to exogenous RNA molecules emphasizes the oral route of exposure as the relevant route of exposure to RNAs in foods, including those that confer traits to GE crops. The oral route is also of key importance to the potential impact of any RNA residues in foods resulting from topical uses of nucleic acids in an agricultural setting. Based on the history of safe consumption of RNA molecules in the diet (including dsRNAs with sequence identity to the consuming organism) and the biological barriers detailed above, the weight of the evidence suggests that ingested nucleic acids are neither absorbed to a significant extent nor capable of triggering a biological response in a consuming organism. This is largely due to the collective impact of each of the individual biological barriers discussed above, leading to a significant reduction in dsRNA levels in terms of the amount available for possible functional activity relative to the amount ingested; these barriers result in insufficient copies being available within a given cell to mediate biological function (White, 2008; Snow et al., 2013; Witwer, 2016). Plant miRNA uptake in mice is quite limited (i.e., less than one copy per ten cells) and more importantly, is insufficient for mediating RNAi in the ingesting organism, which requires at least 100 copies of RNA per cell (Snow et al., 2013). The number of RNA copies per cell needed to mediate biologically meaningful RNAi may be as many as 1,000–10,000 copies (Title et al., 2015). Plant small RNAs are also bound tightly to Argonaute proteins within the RISC complex as necessary for their function and they are not known to either freely dissociate from these complexes or undergo uptake and exchange (either free or complexed) into functional host Argonautes in order to engage targets in a consuming organism (Witwer, 2016). Therefore, these barriers and limited uptake along with the inability of exogenous plant RNAs to function in a consuming organism severely limit the possibility of diet-derived small RNAs having activity in the ingesting organism.

The concept of potential uptake and activity of dietary small RNAs was evaluated in the context of rice miRNAs. In a 2012 peer-reviewed publication, it was suggested that a specific miRNA in rice (miR168a) was absorbed into the bloodstream of ingesting mice and into systemic tissues where it reduced levels of a targeted protein (LDL Receptor Adaptor Protein 1, LDLRAP1) and mediated downstream physiological impacts on cholesterol levels (Zhang et al., 2012). Possible explanations for the findings of Zhang et al. (2012) include laboratory contamination and very low level detection, leading to false positives within PCR-based measurements of RNA uptake (Witwer et al., 2013; Lusk, 2014; Tosar et al., 2014) and from issues surrounding the experimental design of the feeding studies, e.g., diets were not nutritionally balanced (Dickinson et al., 2013). After a 12-h fast, Zhang et al. (2012) fed mice a carbohydrate rich diet of 100% raw rice (contains miR168a) for several days prior to observing dysregulation of a cholesterol related protein and serum LDL cholesterol levels. When diets abundant in miR168a were fed to mice following a 2-week washout period of feeding on a synthetic diet (no plant material or rice-derived miR168a), the same serum cholesterol impacts of diet feeding were observed as those of Zhang et al. (2012), but only when nutritional equivalence of the test and control feeding regimen was not maintained (e.g., only when a diet of mostly rice was given) (Dickinson et al., 2013). Such differences in cholesterol were not observed when a miR168a-rich but nutritionally balanced diet was administered following the washout period. No apparent absorption of miR168a was observed in the blood or tissues of mice in this feeding study (Dickinson et al., 2013). No modification of the LDLRAP1 target protein expression levels were observed by Dickinson et al. (2013) under any experimental conditions using a mouse-specific ELISA assay run at several dilutions, indicating that Western blotting conducted by Zhang et al. (2012) may not have accurately reflected impacts of dietary miRNAs on protein levels. Therefore, dietary miR168a does not undergo absorption to a biologically meaningful extent following rice feeding and any levels of absorption are insufficient to modulate gene expression levels in the consuming mammal.

When droplet digital PCR was leveraged to evaluate miRNA uptake from feeding experiments in rhesus monkeys, uptake from the diet was not evident over a time course (Witwer et al., 2013). This PCR method allows for many PCR reactions that collectively result in identification of false-positive amplifications. Subsequent publications have demonstrated that contamination of PCR reactions can lead to false-positive amplifications within biological samples (Tosar et al., 2014). An elegant study using knockout mice for specific miRNAs demonstrated that mice do not take up dietary miRNAs in sufficient quantities to mediate gene suppression (e.g., less than one copy per cell detected), as evaluated through feeding these same miRNAs to knockout mice (Snow et al., 2013). This study by Snow and colleagues also conducted fruit feeding studies in healthy humans and were unable to detect fruit-derived miRNAs after measuring them in fruit and looking for them in blood following consumption. This calls into question the detection of exogenous RNAs in the bloodstream of humans and other mammals in laboratory and sequencing studies (Wang et al., 2012; Zhang et al., 2012; Tosar et al., 2014; Bagci and Allmer, 2016; Kang et al., 2017) and the role of contamination in these analyses, as detected miRNAs have included sequences derived from microbes, yeast, insects, worms, rodents, and foods for which plausibility of exposure is not apparent (i.e., rat miRNAs in human samples; carrot, cabbage, and sorghum miRNAs in mouse samples). Microbial sequences in the bloodstream could indicate sepsis and the presence of exotic RNAs in the bloodstream from the diet seems implausible with both being indicative of contamination. This has been extensively evaluated in 800 human data sets (Kang et al., 2017), supporting the conclusion that contamination and not dietary uptake is the most plausible explanation for widespread and/or abundant detection of exogenous miRNAs in mammalian blood samples.

Despite substantial evidence from well-controlled feeding studies in rodents and humans that calls into question the ability for RNA from the diet to undergo significant uptake and have putative activity in mammals (Dickinson et al., 2013; Snow et al., 2013; Witwer et al., 2013), the dietary RNA uptake hypothesis has continued to be explored. This research has resulted in a number of feeding studies claiming RNA absorption from the diet through various plant sources (Liang et al., 2015; Yang et al., 2015a, 2016, 2017) and potential physiological impacts including papers claiming the ability of dietary RNAs to treat highly infectious viruses (Zhou et al., 2015), regulate intestinal growth (Li et al., 2019), and treat cancer (Mlotshwa et al., 2015). Some experimental issues with these studies (as described in detail by Petrick et al., 2016a) call into question whether these papers indeed indicate the ability of typical dietary RNAs (e.g., miRNAs) to impact gene expression in a consumer (Petrick et al., 2016a). For example, Zhou et al. (2015) and several of the above papers by Yang and colleagues evaluated putative biological impacts of a highly thermostable and GC-rich (85% GC content) ribosomal RNA fragment termed “MIR2911” (other RNAs in the fed fraction were degraded by heat during preparation), which is not a miRNA and its properties are not reflective of a typical dietary miRNA. Furthermore, this RNA was reported by Zhou and colleagues to have potent antiviral activity (reduction in viral titer, reduction in body weight loss, and increased survival) despite its low level in the diet. These data contradict years of pharmaceutical research regarding lack of oral RNA efficacy and claims of biological activity of orally administered/ingested RNAs have been presented in only a small number of papers to date (Zhang et al., 2012; Mlotshwa et al., 2015; Zhou et al., 2015; Li et al., 2019), with these reports remaining unconfirmed.

Li et al. (2019) noted that after feeding corn containing diets to mice, an RNAi-mediated mechanism impacts several genes and their targeted proteins. However, the *in vivo* data demonstrate modest changes in relative protein levels of approximately 1–3 fold (by Western blot with anti-human antibodies) in a small sample size (*n* = 4) at a single time point (7 days). Without a deeper understanding of the comparative nutritional components of the fed diets (e.g., comparison of fat, protein, carbohydrates, and key nutrients across the diets) and evaluation of the normal range of expression variability of the evaluated target proteins, along with thorough histopathological assessment of these animal intestines, the physiological relevance of the slight changes in mRNA and protein levels and the noted changes in morphology following corn feeding is difficult to assess. Furthermore, the results of this paper are inconsistent with results demonstrating no uptake of corn miRNAs into the bloodstream of mice after 2 weeks of oral dosing (Huang et al., 2018).

The hypothesis that “you are what you eat” and that nutrition may serve as a therapeutic modality is an attractive one. This may explain in part why there have been many reviews on the topic of dietary miRNA uptake and/or activity in mammals, often with a theme of dietary miRNAs being potential mediators of our responses to foods and also reviews that challenge this concept (Cottrill and Chan, 2014; Witwer and Hirschi, 2014; Hirschi et al., 2015; Yang et al., 2015b, c). The result of these reviews has been a great deal of interest in the subject, however, the primary literature leveraged in these reviews lacks robust evidence that any of the reported uptake and activity of dietary miRNAs results in physiologically meaningful impact to the consuming organism or adverse impact to animals following consumption.

There have been two published 28-day repeated-dose oral toxicology studies looking at high doses of insecticidal double-stranded RNA sequences fed to mice (Petrick et al., 2015, 2016a,b). Following 28-days of repeat oral dosing of mice at doses of ≥48 mg/kg body weight with siRNAs or a long dsRNA with 100% sequence complementarity to mouse vacuolar ATPase (gene target provides corn rootworm control when rootworm sequence is expressed in corn), no treatment-related toxicity or target gene suppression was observed. When a corn rootworm active RNA sequence (240 base pair active dsRNA embedded in a 968 nucleotide RNA) was fed to mice at doses of up to 100 mg/kg body weight, no treatment-related effects were observed (Petrick et al., 2016a). Therefore, the no-observed adverse effect level was 100 mg/kg body weight (the highest dose tested), a dose that is estimated to be at least 2.5 billion times higher than mean per capita maize consumption in Europe and the United States (Petrick et al., 2016a, b). Based on the weight of the evidence from mammalian toxicology studies, ingested RNA molecules do not undergo physiologically meaningful uptake and do not present a hazard to humans following ingestion.

Potential for uptake and impacts of exogenous dsRNAs following ingestion have been considered by regulatory authorities. The European Food Safety Authority (EFSA) noted that, “Based on the current knowledge, gained in pharmaceutical research and development, RNAi molecules show limited bioavailability, quick turn-over (for further reading please refer, for example, to Ballarín-González et al., 2013) and no adverse effects following oral gavage (even for formulations specifically designed to maximize their effects).” (Ballarín-González et al., 2013; EFSA, 2014). A Scientific Advisory Panel held by the US EPA (USEPA, 2016) noted that, “there are no reliable evidence [*sic*] that exogenous dsRNAs are taken up from the gut into mammalian circulation to exert its functions in the ingesting organism.” Furthermore, the panel considered such impacts unlikely due to arguments concerning stoichiometry, noting the low levels of blood concentration relative to those needed to induce regulation of gene expression. Food Safety Australia New Zealand considered this topic and concluded that, “A history of safe human consumption of RNAi mediators exists, including those with homology to human genes. The evidence published to date also does not indicate that dietary uptake of these RNAs from plant food is a widespread phenomenon in vertebrates (including humans) or, if it occurs, that sufficient quantities are taken up to exert a biologically relevant effect (FSANZ, 2015).” Based on the weight of the evidence from mammalian studies and regulatory considerations, ingestion of RNA molecules does not present a hazard to humans or other mammals.

## Published Studies in Non-Mammalian Vertebrates

It still remains unknown whether all the factors required to initiate RNAi from ingestion of exogenous dsRNAs exist in non-mammalian vertebrates. Sifuentes-Romero et al. (2011) reviewed several studies of the RNAi response in a variety of animals, including frogs (*Xenopus laevis*), fish (*Danio rerio*, *Oncorhynchus mykiss*, *Cyprinus carpio*), chicken (*Gallus gallus*), and turtle (*Trachemys scripta*) and concluded that there is enough evidence to support an effective, potent, and reproducible RNAi response in these organisms using invasive delivery techniques, such as microinjections and electroporation. Similar responses could also be observed in birds (*Zonotrichia leucophrys gambelli*) when siRNAs were directly administrated into their brains (Ubuka et al., 2012). In Sea lampreys (*Petromyzon marinus*), gene suppression and phenotype effects were observed via injection of siRNA into embryos. Larvae of lampreys also showed gene suppression after feeding on a siRNA with a liposome-based formulation as the transfection reagent, however, feeding naked siRNA (without transfection reagents) failed to induce a response (Heath et al., 2014). These data all confirm the feasibility of RNAi technology as a tool for conducting fundamental biological process studies and loss-of-function experiments in a variety of organisms and not only model systems. However, to date, there is a lack of evidence of RNAi effects in vertebrates orally exposed to naked dsRNA. Bachman et al. (2016) carried out an extensive study on the effects of dsRNA on several organisms as part of the ecological risk assessment for DvSnf7, a dsRNA-based PIP (Bachman et al., 2016). Included in the study, a corn rootworm active dsRNA (1000 μg dsDvSnf7/kg diet) was incorporated into the diet of Bobwhite quail (*Colinus virginianus*) and the animals were observed for 14 days. Body weights, signs of toxicity, abnormal behavior, and mortality were recorded, and no adverse effects were observed, indicating an absence of non-specific RNAi responses (Bachman et al., 2016).

## Human Health Risk Considerations for RNA-Based Biopesticides

Pesticides are an indispensable tool for farmers and are used as an efficient and beneficial tool for pest management in most sectors of agricultural production. However, there are always hazards and associated risks associated with the exposure of farmers and/or professional applicators when mixing and applying the product or working in treated fields (Damalas and Koutroubas, 2016), and for the general public in the case of residues in food and drinking water (Damalas and Eleftherohorinos, 2011). The risk of a pesticide to any living organism is assessed by estimating its associated hazards or potential to cause harm (due to the inherent toxicity of a particular substance) and the possibility of exposure (Damalas and Koutroubas, 2016). When exposure occurs, both the exposure amount (dose) and duration (length and frequency) are important in understanding potential risks associated with pesticide toxicity (Frank and Ottoboni, 2011). Therefore, risk assessment is a comprehensive evaluation of the pesticide toxicity profile and an assessment of exposure. Pesticide labels contain not only information regarding the potential hazards of the product but also use requirements that reduce potential exposures. When agricultural chemical products are used in accordance with the label instructions, even toxic substances can be applied with relatively low risk.

### Other Routes of Exposure

Exposure is required in order for any risk to exist. For humans, there are several different possible routes of exposure to dsRNA-biopesticides. Accidental oral exposure or residues in food and water represent two possible scenarios for ingestion and the potential risks associated with these exposures has been discussed above. Other possible exposure routes to consider are through dermal absorption and inhalation, potentially relevant routes for occupational exposure (Maroni et al., 1999).

#### Dermal Absorption

It is appropriate to consider potential dermal exposure to agricultural products that may be deployed in or applied to the field. In the case of dsRNA molecules, the scientific literature demonstrates that they undergo limited dermal uptake. Nucleic acids are potential therapeutics for various diseases due to their specificity, and delivery through the topical route would be desirable for drug developers. However, there are well known delivery challenges for these therapeutics due to biological barriers such as nuclease degradation, rapid clearance from the bloodstream, and poor bioavailability- barriers that remain challenges for systemically delivered nucleic acid therapeutics (Rayburn and Zhang, 2008; Pecot et al., 2011). Several challenges exist for topical delivery of nucleic acids despite advantages over intravenous and oral delivery. For example, topical delivery avoids enzymatic degradation in the bloodstream, lowers systemic toxicity potential and provides sustained and controlled delivery (Brown et al., 2006). A formidable barrier to absorption is posed by the stratum corneum, a thin layer of anucleated corneocytes held tightly together by a lipid matrix that forms the outermost layer of skin, and serves primarily as a barrier to foreign materials (Zakrewsky et al., 2015). If foreign materials are to cross the stratum corneum, this must occur either through diffusion via lipid channels and/or transcellular passage through corneocytes, or via entry through sweat ducts or hair follicles (Zakrewsky et al., 2015). Transport within the lipid bilayers is the most common of these routes; however, this route excludes most foreign materials. Large hydrophilic molecules (short dsRNAs are >10 kD) undergo negligible transport across the skin without transport enhancers or various cellular membrane disruption techniques (e.g., microporation or electroporation) (Zakrewsky et al., 2015). Even in the very unlikely scenario of significant dsRNA absorption and systemic distribution following topical exposure, such RNAs would be expected to undergo rapid metabolism and clearance and would be subjected to the numerous biological barriers discussed above. Therefore, rapid breakdown and clearance, along with various dermal barriers to macromolecules (e.g., exogenous dsRNAs) greatly limit the potential for dermal toxicity of dsRNA molecules.

#### Inhalation

Most RNAi-based biopesticide products will be applied using similar methods as traditional chemical pesticides (e.g., spray applications), therefore, respiratory exposure should be considered as another potential exposure route. During a pesticide spray application, a significant portion of the product may not reach the intended target and may be transported to other areas through spray drift (van den Berg et al., 1999; Degrendele et al., 2016), which can potentially be inhaled and deposited in the human respiratory system. However, the pulmonary deposition of particles in the lung is dependent on the aerodynamic diameter of the inhaled droplets (Hinds, 1982). Most agricultural particles are large enough that they are not deposited in the lung, but rather, are cleared from the upper respiratory tract, resulting in a secondary oral exposure rather than pulmonary exposure (Sherman et al., 2015).

Challenges with the development of inhaled RNA therapeutics demonstrate how challenging it is to use this route to effectively deliver RNA molecules in humans. Some recent inhaled RNA therapeutic studies have shown advances in the delivery of RNAi-based drugs to the lungs (reviewed by Youngren-Ortiz et al., 2017; Thanki et al., 2018). However, a common conclusion across these studies is the requirement of the design and development of specifically engineered formulations to safely and effectively deliver the RNAi-based drug. The engineered molecules must overcome the existing biological barriers, such as degradation by RNase, mucociliary clearance, clearance by impaction and coughing, and alveolar macrophage clearance (Youngren-Ortiz et al., 2017). Adding to that, there is the challenge of developing suitable devices for pulmonary administration of inhalable RNAi-based therapeutic formulations (Thanki et al., 2018).

As with any pesticide, when spraying, one must take routine precautions to prevent inhalation of dsRNAs by applicators, for example, through the use of appropriate personal protective equipment (PPE). At present, there seems to be an absence of published data concerning the potential biological impact of inhalation of RNA molecules. Given that these can potentially be immunostimulatory molecules (via non-oral routes) per the literature, a non-sequence specific inflammatory response may occur upon significant exposures, hence the recommendation to use the appropriate PPE to limit inhalation exposures for spray applications as with any other sprayed crop protection product.

### Different RNA Structures and Formulations

It is widely known that different species respond differently to environmental dsRNA (Rodrigues and Figueira, 2016; Wang et al., 2016; Christiaens et al., 2018a). In insects, for instance, lepidopterans are considered recalcitrant to naked dsRNA via oral delivery (Terenius et al., 2011; Shukla et al., 2016; Guan et al., 2018). In order to apply RNAi technology to manage non-responsive pests, several companies have developed different RNA structures as carriers to improve the delivery of dsRNA. In addition, because of its intrinsic structure, RNA forms can also be naturally modified to form several structures, such as supercoiled or in a hairpin. However, all forms of RNA structures, whether modified to increase the responsiveness of the target pest or occurring naturally, are likely to be degraded through the digestive process by the combination of RNases and acids found in the human digestive system (USEPA, 2014).

Another approach to enhance RNAi response in some pests is the development of formulations (Christiaens et al., 2018b; Dhandapani et al., 2019) aiming to improve delivery and availability of the dsRNA to the target. However, considering the complexity and the multiple biological barriers present in mammals (already discussed herein), it is unlikely that formulated RNAi-based products developed for agricultural uses will efficiently deliver dsRNA into human cells following ingestion. In support of this, researchers have developed several formulations to address the delivery and biostability of RNAi inside the human body and clinically relevant responses have been limited to injected drugs, such as vaccines and cancer therapy drugs (Ji et al., 2011; Kang et al., 2011; Lin et al., 2013; Cavallaro et al., 2017). O’Driscoll et al. (2019) reviewed the progress and feasibility of oral delivery of RNA-based drugs and have observed a lack of clinical trial data; indicating, “while progress has been made through innovative formulation strategies to date clinical translation of oral products has not been realized” (O’Driscoll et al., 2019).

The wide variety of substances and technologies that can be developed and optimized into a cost-effective formulation to enhance dsRNA delivery and stability in the field can be a reality in the future. The recommendation raised during the OECD meeting to mitigate this risk is to consider each new formulation individually in a case-by-case assessment.

### Non-specific Effects (Innate Immune System or RNAi Machinery Saturation)

Exogenous dsRNAs are known to stimulate the innate immune response (e.g., the interferon pathway) under experimental conditions permitting high levels of RNA exposure (Judge et al., 2005; Robbins et al., 2009; Jackson and Linsley, 2010). As reviewed by Petrick et al. (2013), such induction of the innate immune system has been characterized using *in vitro* systems that leverage transfection reagents and high RNA concentrations, and in some cases, in animal models. These responses are mediated via receptors that interact with dsRNA such as the Toll-like receptors (TLR3, TLR7, TLR8), and enzymes including the dsRNA binding protein kinase PKR, and the RIG-I and MDA-5 RNA helicases (Robbins et al., 2009). In animals, this induction of the innate immune response appears to be constrained to injection/systemic exposure to RNAs (Robbins et al., 2009) and there is no apparent evidence in the literature indicating that this occurs following oral exposure, the most relevant human exposure route for risk assessment considerations of agricultural uses of externally applied dsRNA.

In a set of mouse and blood cell studies from the pharmaceutical industry, sequence-dependent stimulation of the innate immune response in mice was demonstrated following intravenous delivery of dsRNAs (siRNAs) encapsulated in liposomes (Judge et al., 2005). However, this response did not occur with naked RNAs. Judge and colleagues note that delivery vehicles were required for immunostimulation and that encapsulated RNA formulations are protected from nucleases yielding extended circulation times relative to those not encapsulated. This dependency on delivery vehicles may stem from their ability to deliver RNA to the endosomal compartment of the cell that houses RNA-sensing pattern recognition receptors that facilitate an immune response (Robbins et al., 2009; Jackson and Linsley, 2010). Induction of the innate immune system was not observed with the injection of unmodified naked siRNAs (Heidel et al., 2004; Judge et al., 2005). An oral siRNA study using a specialized delivery vehicle to promote absorption did not show evidence of immunostimulation even in the presence of target gene suppression (Aouadi et al., 2009). Therefore, it is apparent that immunostimulation by dsRNAs appears to require specific delivery routes (e.g., injection), delivery vehicles, specific sequence motifs, stabilizing modifications, and significant exposures, all of which have limited or no relevance to dietary exposures that may occur from agricultural uses of dsRNA.

Although a review article has indicated immunostimulation as a possible hazard from oral exposure to dsRNAs in agriculture (in this case, from GE crops; Lundgren and Duan, 2013), exposure scenarios required for observation of immunostimulation (e.g., injection of high doses of nucleic acids) are not relevant to environmental or dietary exposures that could be encountered by humans or non-target mammals through exposure to exogenous RNAs. This is because immunostimulation from an ingested RNA would require absorption of a sufficient concentration for induction of the response, a phenomenon that is improbable given the multitude of biological barriers to the attainment of significant levels of systemic RNA after dietary consumption. DvSnf7 RNA, a corn rootworm active RNA molecule when expressed in corn plants (includes a 240 bp insect active dsRNA) was safely administered to mice via oral gavage for 28 days without any apparent clinical or toxicological signs of immunostimulation or immune response at a dose of up to 100 mg/kg (Petrick et al., 2016a). This dose is 2000 times higher than the 0.05 mg/kg cited as capable of inducing potent cytokine responses following dsRNA injection into the mouse (Judge et al., 2005). Furthermore, this 100 mg/kg dose is 50 times higher than the 2 mg/kg experimental intravenous siRNA dose used by Judge and colleagues that produced immunostimulation for formulated siRNAs but not naked siRNAs. The lack of oral immunostimulation by dsRNAs is further evidenced by the extensive history of safe consumption of RNAs in the diet from food, be they short or long dsRNAs (Fukuhara et al., 2006; Ivashuta et al., 2009; Jensen et al., 2013; Petrick et al., 2013; Frizzi et al., 2014), owing to the lack of appreciable absorption or systemic tissue exposures following ingestion.

Another putative risk from dsRNA in GE crops mentioned by Lundgren and Duan (2013) is the saturation of the RNAi machinery. In one of the seminal papers on RNAi machinery saturation, this phenomenon occurs *in vitro* in a dose-dependent manner after transfection of relatively high doses of small RNAs into cultured cells (Khan et al., 2009), exposure conditions that are not relevant to environmental or dietary exposures to exogenous dsRNAs. This phenomenon occurs as a result of a limited number of RISC complexes being overwhelmed by the amount of externally applied small RNA, under supraphysiological conditions. As reviewed by Jackson and Linsley (2010), other papers demonstrating RNA machinery saturation relied on “sustained high-level expression of [short hairpin] shRNAs in the liver of adult mice,” a transgenic approach relying on over-expression of a short RNA hairpin in a mouse. This phenomenon was also reviewed by Grimm (2011). In contrast to these saturating doses, another study looking at *in vivo* delivery of exogenous RNAs achieved about 80% silencing of targeted transcript without affecting cellular miRNA biogenesis or function, e.g., this silencing did not result in saturation of the RNA machinery (John et al., 2007). Indeed, Lundgren and Duan do not seem convinced of the biological relevance of RNAi machinery saturation as their review stated that, “it is unclear how dsRNAs produced by plants could affect the RNAi machinery used by both target and non-target organisms and whether there will be sufficient small RNA produced by GM plants to saturate an organism’s cellular machinery (Lundgren and Duan, 2013).” Questions on sufficiency of exposure are equally applicable to externally applied “topical dsRNA” due to limited levels of application in the field (i.e., applications to be limited by cost of goods) and extensive barriers to exogenous dsRNAs in non-target mammals. Therefore, the plausibility of systemic dsRNA exposure from agricultural applications at levels capable of initiating RNAi machinery saturation is highly questionable. Toxicological studies in mammals (discussed above) exhibited no adverse findings at oral dose levels up to billions of times anticipated human dietary exposure, providing strong support for the implausibility of RNAi machinery saturation as a potential source of adverse effects from exogenous dsRNA exposures in mammals.

## Conclusion

The data available indicate that significant systemic absorption of intact dsRNA following dietary exposure of RNAi-based biopesticides is highly improbable in humans and other vertebrates. The longstanding history of safe consumption of dsRNAs naturally found in all foods and feeds, including those with complementarity to human and vertebrate transcripts, supports the safety of these molecules for use as biopesticides. The principal reason for the lack of biological response to exogenous dsRNAs is the presence of multiple biological barriers at the gastrointestinal, bloodstream, and cellular levels in mammals. Even in the very unlikely scenario of significant dsRNA absorption and systemic distribution following exogenous exposure during the product application, such RNAs would be expected to undergo rapid metabolism and clearance. Owing in part to the history of safe consumption and the favorable toxicity profile of exogenous dsRNA molecules in mammals (including insecticidal sequences), biological barriers, and their fate *in vivo*, these biological macromolecules should not be presumed to be inherently more risky than conventional small molecule agrochemicals. Regulatory authorities have not yet established standard procedures for assessing dsRNA-based agricultural products. The safety assessment of each of these products is currently considered on a case-by-case basis by these authorities. However, the existing robust regulatory framework for small molecule agrochemicals is applicable as a general framework for conducting risk assessment of dsRNA-based agricultural products. As with any emerging technology, the regulatory framework will continue to evolve; however, the experience with the review of dsRNA-based GE crops has demonstrated that the existing regulatory paradigm for biologically based crop protection products is adequate for this mode of action. The OECD Conference, along with this paper, increase clarity on both hazard identification and potential risks of RNAi-based biopesticides while also promoting important dialogues among different stakeholders to help facilitate the exchange of ideas between them.

## Author Contributions

TR and JP contributed equally to draft the work and revised it critically for important intellectual content.

## Footnotes

1 https://www.oecd.org/chemicalsafety/pesticides-biocides/conference-on-rnai-based-pesticides.htm
